# Evaluating the impact of COVID-19 pandemic on the physicians' psychological health: A systematic scoping review

**DOI:** 10.3389/fmed.2023.1071537

**Published:** 2023-03-28

**Authors:** Shaista S. Guraya, Prianna Menezes, Isabell Nelson Lawrence, Salman Yousuf Guraya, Fiza Rashid-Doubell

**Affiliations:** ^1^School of Medicine, Royal College of Surgeons Ireland – Medical University Bahrain, Muharraq, Bahrain; ^2^NHS Grampian, Aberdeen, United Kingdom; ^3^Clinical Sciences Department, College of Medicine, University of Sharjah, Sharjah, United Arab Emirates

**Keywords:** COVID-19, physician, psychological health, personal wellbeing, professionalism

## Abstract

**Background:**

COVID-19 has endangered healthcare systems at multiple levels worldwide. Published data suggests that moral dilemmas faced during these unprecedented times have placed physicians at the intersections of ethical and unethical considerations. This phenomenon has questioned the physicians' morality and how that has affected their conduct. The purpose of our review is to tap into the spectrum of the transforming optics of patient care during the pandemic and its impact on psychological wellbeing of physicians.

**Methods:**

We adopted the Arksey and O'Malley's framework, defining research questions, identifying relevant studies, selecting the studies using agreed inclusion and exclusion criteria, charting the data, and summarizing and reporting results. Databases of PubMed/Medline, Web of Science, Scopus, Science Direct, CINAHL, and PsycInfo were searched using a predefined search string. The retrieved titles and abstracts were reviewed. Later, a detailed full-text analysis of the studies which matched our inclusion criteria was performed.

**Results:**

Our first search identified 875 titles and abstracts. After excluding duplicates, irrelevant, and incomplete titles, we selected 28 studies for further analysis. The sample size in 28 studies was 15,509 with an average size of 637 per study. Both quantitative and qualitative approaches were used, with cross-sectional surveys being utilized in all 16 quantitative studies. Using the data from semi-structured interviews, several discrete codes were generated, which led to the identification of five main themes; mental health, individual challenges, decision-making, change in patient care, and support services.

**Conclusion:**

This scoping review reports an alarming rise in psychological distress, moral injury, cynicism, uncertainty, burnout, and grief among physicians during the pandemic. Decision-making and patient care were mostly regulated by rationing, triaging, age, gender, and life expectancy. Poor professional controls and institutional services potentially led to physicians' crumbling wellbeing. This research calls for the remediation of the deteriorating mental health and a restoration of medical profession's advocacy and equity.

## Background

Corona virus disease 2019 (COVID-19) pandemic has generated a widespread distress in the general population and particularly among the frontline physicians (1). The WHO has voiced grave concerns about the mounting emotional toll and deteriorating mental and physical wellbeing of physicians during the COVID-19 pandemic worldwide (2). Similarly, the National Health Service, UK has reported a staggering rise in the emotional burdens and levels of anxiety among physicians, which may adversely affect their decision-making abilities and professional conduct and behaviors (3). The already overstretched, understaffed and under resourced healthcare systems have succumbed to the ever-increasing pressure and public expectations to continue working optimally during the pandemic (4).

The physicians' psychological health and professional and personal life has been challenged during the pandemic by extra duty hours, workload burden and the fear of being infected in health-care facilities (5). In addition to the challenges on physicians' mental and physical wellbeing, a constellation of factors including stress, anxiety, depression and burnout during the COVID-19 outbreak have contributed to the deterioration in psychological health (6). These include, but are not limited to, personal worries, uncertainty, lockdowns and closures, and reduced social contact (7). Concomitantly, the surge in demand for all sections of medical care and the re-allocation of scarce health care resources by minimizing the level of care for other patients have raised several ethical concerns of equity and social justice (8).

During the unprecedented crisis, the complex matrix of personal, professional, and societal expectations for physicians has substantially undermined their confidence and performance. The physicians are facing issues about professional accountability and moral obligations, mostly due to a lack of guidance and legislative controls which would have given them the ability to choose between alternatives (9). In this perspective, the literature has reported an escalating rise in various forms of psychological distress in physicians including moral injury which “results from actions, or the lack thereof, that violates someone's moral or ethical code” (10, 11). A range of experiences constitute moral injury such as a perception of guilt, shame, revulsion, and animosity.

The compounding impact of psychological disconnect, frustration, compromised decision-making, resource relocation and working under pressure is believed to have adversely affected the physicians' personal and professional wellbeing during the COVID-19 outbreak (12). In addition, leisure time physical activity as well as physical inactivity can potentially attribute to the stagnant lifestyle. There is a dire need for concerted efforts to mitigate the impact of modifiable risk factors which can potentially sabotage the physicians' wellbeing and their ability to cope with the unprecedented crisis (13). Unfortunately, though an existing body of literature has reported some evidence of issues related to the physicians' psychological distress and their personal and professional predicaments, there remains a paucity of understanding on this topic. The objective of this review was to determine the impact of COVID-19 on psychological, personal, and professional wellbeing of physicians. Additionally, this research aimed to provide a clear remedial pathway that can potentially navigate the professional and personal wellbeing of frontline physicians.

## Methods

The search design of our systematic scoping review was based on the following stages as described by the Arksey and O'Malley's framework (14).

- Defining the research questions based on the context of scoping review using a triad of participants, concept, and context.
- Identifying relevant studies that matched the defined research questions and purpose.
- Selecting studies using the pre-determined inclusion and exclusion criteria.
- Charting the data.
- Summarizing and reporting the results.

Our search strategy did not apply the optional sixth requisite of Arksey and O'Malley's framework, the necessity of consultation with potential stakeholders, as this was not the main purpose of our research.

In the following sections, we have detailed each step of the Arksey and O'Malley's framework as used in our study.

### Defining research questions

- How has COVID-19 affected the psychological health of physicians involved in the care of patients?
- What are the effects of the COVID-19 on the personal and professional wellbeing of physicians?

### Identifying relevant studies: Databases and process of data selection

On June 17, 2021, the principal investigator (PI) (SSG) searched six databases: PubMed/Medline, Web of Science, Scopus, Science Direct, CINAHL and PsycInfo. PubMed/Medline was the mainstay to systematically develop a search string, which was later extrapolated to other databases. We used ((Physicians) AND ((((Covid 19) OR (Sars CoV2)) OR (acute respiratory distress syndrome covid-19)) OR (2019-nCoV))) AND ((((moral injury) OR (moral distress)) OR (moral conflict)) OR (ethical dilemmas)) OR (psychological wellbeing) OR (physician and personal well-being)) terms and text words for the English language original articles published between January 2020 and June 2021. Original articles with quantitative, qualitative, and mixed methods study design were included. All selected keywords were searched using “abstract” and “article title” (alternatively “topic”) and in the Medical Subject Headings (MeSH) terms or Thesaurus, where available. No filters or limitations were applied to retrieve the largest number of results. The articles published outside the defined time window, review and editorial articles, personal views and commentaries were excluded. Search for gray literature was conducted in the ProQuest Dissertation and Thesis. A full search log, including detailed search strings for all included information sources, results and notes are available in Appendix I.

### Selecting the studies: Data extraction

We used the Preferred Reporting Items for Systematic Reviews and Meta-analyses (PRISMA) guidelines for data mining and for the selection of studies for our scoping review (15). The three main constructs of our review included: *concept* (moral injury, moral distress, moral conflict, ethical dilemmas, medical professionalism), *context* (COVID-19) and *participants* (physicians involved in COVID-19 care) using the pre-determined inclusion and exclusion criteria. In our scoping review, we followed a structured protocol for the screening and selection of studies and then to review, chart and extract articles using the Covidence v1.0 data extraction template (16). Researchers PM and INL reviewed all titles and abstracts retrieved during the initial search and grouped relevant articles for possible inclusion. To ensure consistency and research quality, SYG reviewed all initially selected titles and abstracts. Any conflicts were resolved by FR-D and SSG. Later, FR-D and SSG reviewed full text of the selected articles. To mitigate research bias, the entire search process was finally reviewed by SSG, FR-D and SYG. We resolved research disagreements and disputes through discussions until a consensus was reached. The final selection of the identified articles was considered for data extraction.

### Charting the data: Data analysis

Each article that qualified for the full-text review was independently reviewed by SSG and FRD. Covidence platform was used for the organization of the descriptive information (e.g., authors, year, type of article, study purpose, research design, participants, and ethical approval). A descriptive-analytical approach was applied for the charting and analysis of the key findings and recommendations emanating from the selected studies (17). FRD, SSG, INL and PM charted the articles separately. Later, all five authors conferred, a consensus was reached, and all overarching key themes were identified.

### Collating, summarizing, and reporting the results: Data synthesis

We performed a descriptive analysis (e.g., year of publication, country, health professionals' disciplines and study purposes) using Excel and performed a thematic analysis by following a grounded theory approach (18). Using induction as a key process, the analyzed data was coded, ideas and potential insights were rendered in the form of a theoretical memo where data was allowed to speak for itself. A constant comparative method was implied which helped in fitting and refitting of categories until the concept maturation was achieved in the form of emerging themes (19). Later those themes and subthemes were further analyzed, discussed, and regrouped until a consensus was reached. Over the course of the analysis, the identified five themes were refined using the hierarchical clustering strategy (20), where we labeled each keyword as a descriptor, and then merged descriptors with maximum similarity into a subtheme. This similarity-based merger continued until all individual descriptors were collated into subthemes.

Though the researchers thoroughly reviewed the literature and assigned special roles for different stages of literature review, the possibility of research bias cannot be completely eradicated. Selection of only English-language articles might have limited a broader perspective of the subject.

## Results

**Figure 1:**
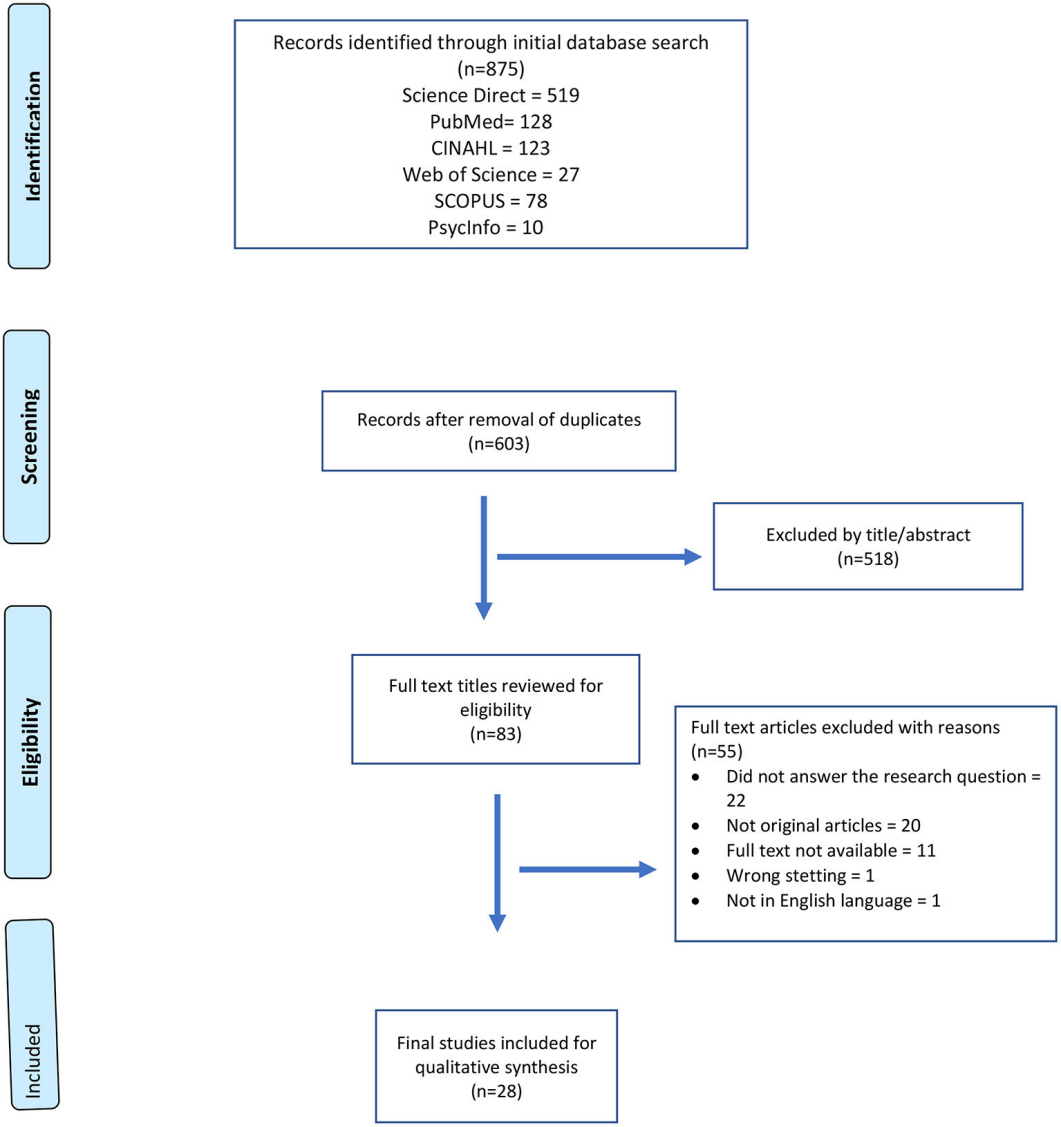
A flow chart for the selection of the studies about physicians' psychological health and personal and professional wellbeing in COVID-19 using the Preferred Reporting Items for Systematic Reviews and Meta-analyses (PRISMA) guidelines.

Our initial search retrieved 875 titles, and after removing 272 duplicates and retaining only English language publications, we found 603 titles for further screening using the abstract analysis. This process further eliminated 518 titles which did not meet the inclusion criteria. A total of 83 full text articles were further reviewed for their eligibility. After full paper review, we included 28 articles in our scoping review for deeper analysis. The entire process using the PRISMA guidelines is illustrated in Figure 1.

**Figure 2:**
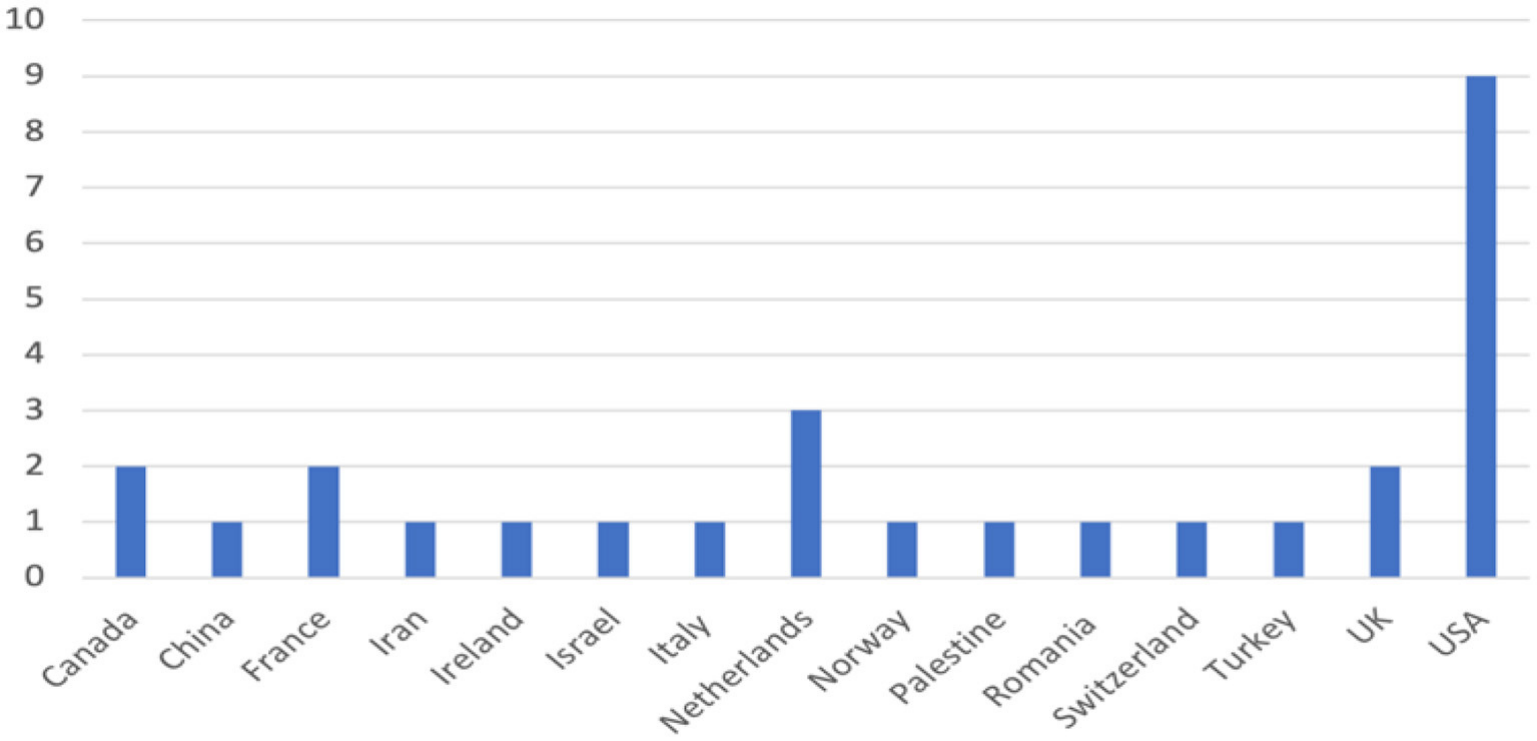
Country-wise representation of the selected studies about physicians' psychological health and personal and professional wellbeing in COVID-19 (*n* = 28).

According to the yearly publication pattern of the selected 28 articles, six articles were published in 2020 and 22 were published until June 17, 2021. Three-quarters of the studies were multi-center (75%), and most of the studies were conducted in hospitals (22/79%), while other sites included hospices, nursing homes and clinics. A graphical representation of the countries of origin of the selected 28 studies is displayed in Figure 2.

**Figure 3:**
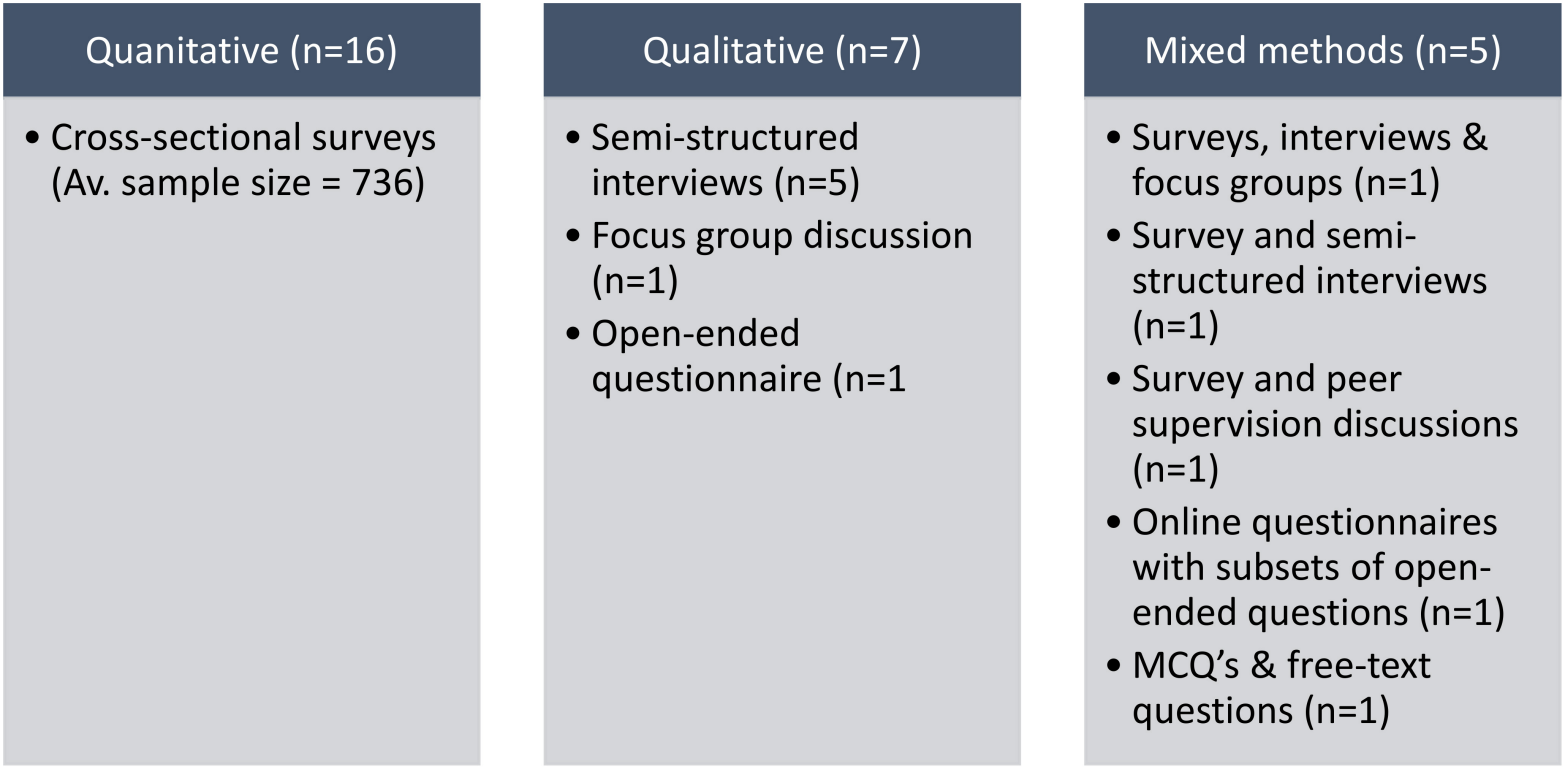
The range of research designs for the collection data in the selected studies about physicians' psychological health and personal and professional wellbeing in COVID-19 (*n* = 28).

Most studies (9/32%) originated in the USA, while other studies were based in the Netherlands (3/11%), United Kingdom, Canada, and France. Quantitative methodology was the most popular research design among the selected 28 studies (16/57%), where the researchers used cross-sectional surveys to acquire data as shown in Figure 3.

Qualitative studies made up the next largest group (7/25%) where semi-structured interviews, focus group discussions and one open-ended questionnaire were used. The remaining five studies (5/18%), used a mixed methods approach for the data collection. For 16 quantitative studies the sample size ranged between 73-3006 with an average of 736, for quantitative limb of 5 mixed-methods studies, this ranged between 13 and 4773 with an average of 1,368 while a smaller number of participants were recruited in qualitative studies, ranging from 22 to 187. A few of the quantitative studies had participants >1000, i.e., 1050 (21), 1500 (22), 3006 (23), 4773 (24) and 1606 (25). It is noteworthy that, out of all survey-based studies, only three studies had a response rate of 50% or greater (3/19%) (26–28), while five did not explicitly report any response rate. Importantly, 27 (96%) studies provided clear ethical statements either by institutional review board approval, exemption, or by stating that ethical approval was not necessary. A breakdown of the descriptive analysis of the physicians' medical subspecialties involved in the research from the selected 28 articles is shown in Table 1.

**Table 1:** Descriptive analysis showing the breakdown of the physicians' medical subspecialties involved in the research from the selected articles (*n* = 28).

**Specialty of practice**	**Number of studies**
Diverse multispecialty	9
Surgical subspecialties	6
Medical subspecialties	11
Intensive medicine	5
Emergency medicine	10
Anesthesia	5
Psychiatry	7
Pediatrics	2
Obstetrics and gynecology	2
Family medicine	2
Radiology	1

In terms of study populations, 10 of the 28 studies did not provide a breakdown of the gender groups. In the remaining 18, the total sample size was 15,509 and women made up almost two thirds of the participants (9,827/63%). A total of seven studies had a primary research objective focused on exploring ethical dilemmas and subsequent decision-making skills of the participants. Other key research objectives of the selected studies in our scoping review are outlined in Table 2.

**Table 2:** Leading research objectives of the selected studies about physicians' psychological health and personal and professional wellbeing in COVID-19 (*n* = 28)^*^.

**Objectives**	**Number of studies**
Explore ethical dilemmas and subsequent decision-making skills	7
Establish impact on quality of patient care	5
Describe the personal experiences, concerns and challenges while working during the pandemic	5
Assess psychological health parameters – burnout, mental health issues	7
Explore correlates of moral injury and distress	4
Identify the stressors that threaten physicians' wellbeing	5
Explore the support structures for promoting physician's emotional wellbeing	4

^*^Some research papers had more than one identifiable key objective.

**Figure 4:**
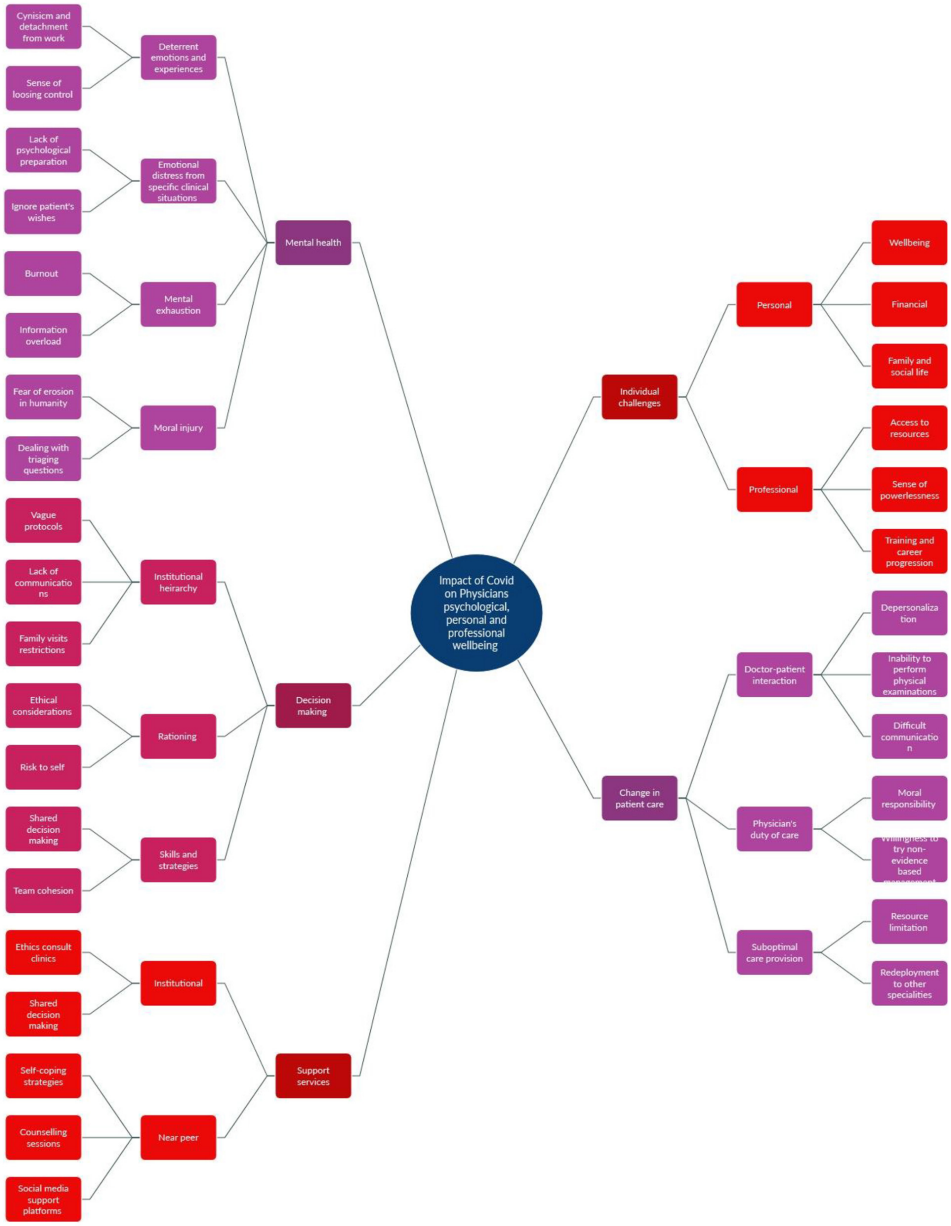
Thematic analysis using a grounded theory approach.

Our iterative review process yielded five themes along with their relevant subthemes; mental health, individual challenges, decision-making, change in patient care, and support services (Figure 4).

Each theme along with its subthemes is elaborated in the following sections. A detailed tabular representation of themes can be found in Appendix II.

## Theme I: Mental health

This theme was identified in (96%) studies and, therefore, we ranked it as the first and the foremost challenge to physicians during the pandemic. There were four relevant sub-themes, deterrent emotions and experiences, emotional distress from a specific clinical experience, mental exhaustion and moral injury. The studies demonstrating an adverse impact of COVID-19 on the mental health of physicians included (21–47). The reviewed body of literature has reported that witnessing patients' sufferings unduly provoked sorrow, grief, and emotional distress among the frontline physicians. This phenomenon prevented them from working in the patient's best interest. Unsurprisingly, several physicians were found to suffer from mental exhaustion, burnout, sleeping disorders, worsening psychological wellbeing and compassion fatigue. Feelings of guilt, insecurity and fear triggered a sense of hopelessness, which led to depression particularly among the physicians dealing with COVID-19 deaths. Cynicism and detachment became typical coping strategies. Others felt lonely, isolated, and experienced a feeling of losing control, bringing about uncertainty which undermined their judgment and confidence.

## Theme II: Individual challenges

This theme was developed from the referred studies; (21–24, 26–30, 32–34, 36–47). There were two relevant subthemes linked to this theme: personal challenges and professional challenges. We found plenty of evidence that during the pandemic, physicians were under tremendous pressure to maintain their personal and professional lives. They experienced sleep deprivation, physical exhaustion, and a decreased quality of life. The family lives of physicians were profoundly disturbed by them staying in hospitals or rental accommodation due to the fear of transmitting COVID-19 to family members. The physicians also faced serious professional challenges owing to feelings of deprivation, powerlessness in decision-making, shortage of personal protective equipment and limited access to testing. Scarce resources and inadequate infrastructure exacerbated the unfair work distribution as did the frequent changes in protocols bringing about both hierarchical and collegial rifts.

## Theme III: Decision-making

The decision-making theme was located in the selected studies; (78.57%), (21, 22, 24–26, 28, 30–41, 43, 48). Our research generated three explicit subthemes of rationing care and triaging decisions, iinstitutional or hierarchical impact on the individual's decision-making outcomes and skills and strategies to improve decision-making. In this research, we found an abundance of evidence that, during the COVID-19 outbreak, the physicians' decision-making became constrained. Triaging decisions and rationing the type of care were based on the patients' age, cognitive status, and the prognosis for survival. The immediacy of life and death decisions without further investigations and management breached ethical principles in the medical field (33). In contrast to the physicians' practice being mediated by the hospital's code of practice, the guidance from institutions became flawed and erratic. Consequently, the core patients' needs such as family visits and patient-doctor consultations were withdrawn, which provoked sentiments of futility and redundancy among physicians. There was some evidence that, to circumvent these challenges to decision-making, the workplace focused on shared multi-disciplinary activity, which contributed toward improved team cohesion under testing conditions.

## Theme IV: Change in patient care

This theme was identified in the referred studies (21–28, 30–41, 44–46, 48). Three subthemes were identified under this theme: suboptimal care provision, doctor-patient interactions, and physicians' duty of care. The body of reviewed literature showed that the provision of healthcare during the outbreak was suboptimal. A paradigm shift in patient care was witnessed. The imposed restrictions were focused on minimizing infection spread, thus resulting in delayed and compromised care. Visitations to the loved ones, considered to be a vital part of the holistic recovery of patients, were reduced or suspended to prevent COVID-19 contamination. Likewise, there was a change in the doctor-patient interactions as they became depersonalized, primarily triggered by the concealment of facial expressions by mask wearing. Furthermore, this concealment deprived the anxious and terminally sick patients of the essential non-verbal facial cues to comfort them (36). Ultimately, with intimacy lessened, the human bond between doctor and patient was lost.

The physicians' duty of care was adversely affected as their actions were impeded by new guidance codes laid down by hospital governance. Leading physicians had to think up creative ways to deliver care such as employing adjunct services to honor patients' wishes and experimenting different strategies that were not based on evidence.

## Theme V: Support services

Finally, support services emerged as a main theme from studies (22, 24, 25, 28, 30, 32–34, 36–40, 43, 46). Two subthemes emanated from this main theme: near-peer support and institutional interventions. In this theme, a wide spectrum of diverse groups formed the near-peer support structures they consisted of friends and family, colleagues, as well as other members of the hospital workforce and multi-professional teams. Although institutional support was in place, unfortunately, it was never sufficient to support the personal and professional wellbeing of physicians. Sporadically, some institutions offered those suffering from post-traumatic stress, anxiety, sleeping disorders and stress-related issues one-to-one support. There was also a myriad of strategies that were hypothetically introduced such as flattened hierarchy, meditation, psychological counseling and rescheduling of the duty hours. However, all these coping strategies lacked commitment, rigor, and sustainability.

## Discussion

This scoping review has reported a substantial impact of COVID-19 disease on the physicians' mental health, individual challenges, decision-making, and patient care. The reviewed body of literature has affirmed that the frontline physicians were subjected to significant stress due to the nature of their duties as they directly engaged with patients infected with COVID-19. The fear of disease transmission to their families, concerns about their own health and of loved ones, being stigmatized and forbidden, and working under extreme pressures have led to physicians' reporting emotional and physical burnouts. The scarcity of resources and redeployment of priorities by healthcare authorities created organizational dissonance and emotional distress and moral injury among the practicing physicians.

The fundamental message, stemming from our scoping review, is that the (re)allocation of resources and priorities have transformed the fiduciary nature of medical professionals' work toward a more utilitarian approach (49). Historically, medical professionals are recognized as moral agents with an inherent mission of justice and responsibility (50). This life-long commitment and accountability cannot be modified or suspended when resources become scarce as the core tenet of medical professionalism centers around the welfare and wellbeing of patients and society. The literature has shown that, during the pandemic, a great majority of physicians lost confidence and professional authority, primarily due to non-medical factors i.e., economic and political decisions (21, 51).

Among the most reported changes in the physicians' mental health was moral distress which entails individuals' reactions to situations when they believe to know the right course of actions but they are unable to do it (52). During the COVID-19 crisis, physicians experienced moral distress when their personal and professional ethos conflicted with their institutional protocols or expectations. One of the adverse manifestations of moral distress is moral injury which was found to be prevalent among the frontline physicians dealing with the coronavirus infected patients (53). Moral injury contains a myriad of element (1) moral dissonance; (2) a sense of guilt, shame and existential conflicts; and (3) the presence of depression, anger, and anxiety (29). During the COVID-19 pandemic, physicians experienced moral injury as they committed unintentional errors leading to mortality or morbidity, due to their inability to prevent harm or death, the transgression of colleagues, supervisors or institutions who clashed with individuals' beliefs, or dealing with leaders who did not take full responsibility for the adverse clinical outcomes (54).

Lu et al. (55) have reported that physicians faced substantially more fear, depression and psychological impairments compared to hospital administrators. This finding shed light on the varying degrees of stress and pressure sustained by different hierarchical strata in the same institution. In their survey-based study by Maftei and Holman (29), using the moral injury events scale, the investigators demonstrated comparably high levels of self-reported negative physical and emotional stress in the COVID-19 and the non-COVID-19 treating groups of physicians. The authors have argued that, regardless of the COVID-19 or non-COVID-19 workforce assignment, medical professionals invariably faced similar workplace related stress, practice similar procedures, and followed medical instructions with a similar rigor and spirit. This review calls for urgent task-specific rehabilitation and remedial programs for the affected medical professionals catering for and supporting their personal and professional realms.

The reported uncertainty and powerlessness of physicians during the pandemic has led to subjective decision-making practices (56). The decision-making theme in our scoping review has thoroughly illustrated that priority settings dilemmas, evolving demands, and uncovered needs have forecasted uncertainty and disbelief among physicians (25). In an interesting study by Idilbi et al., the investigators sought physicians' preferences for the allocation of a ventilator to one of the three COVID-19 infected patients; an 80-year-old man without cognitive illness, a 50-year-old man with Alzheimer's disease (AD) or an 80-year-old man with AD (57). A three-quarters of the respondents placed the 80-year-old man with AD as the last choice, while they were equally divided about the selection of other patients. Similarly, a plethora of research has shown that the priorities of medical care have changed dramatically and key decisions were based on age, gender, ethnicity, life expectancy and associate comorbidities (58). On the same note, rationing, triaging, redeployment of expertise, postponement of non-urgent cases, suboptimal substitutes and a lack of shared decision-making were prevalent in the reviewed literature. Neves et al. (59) have coined this change in practice as “how to decide who lives and who dies?”. From a different perspective, the postponement of elective cases in a wide range of medical and surgical specialties led to unforeseen complications (60).

In this scoping review, we have identified a paradigm change in the pattern of patient care during the COVID-19 pandemic. As reported by Dewar et al. (61) some physicians did not notice any change in their practice, while a vast number of intensivists, ethicists, and general practitioners felt “an entirely new experience” in their clinical work. To combat the crisis, some physicians created alternative management pathways by adopting unorthodox and non-standardized substitutes, which could be harmful and suboptimal (62). Some researchers have argued that, in a desperate attempt to do everything to save lives, sometimes physicians experimented with aggressive treatment options and delayed end-of-life decisions which added distress and frustration among families and society (63, 64). The changing optics of the physicians' approach toward equity and justice especially in the critical care units cannot be overemphasized. Arabi et al., highlighted the pressing need for transformative changes in critical care units by enhancing the ICU bed capacity, redesigning the units for COVID-19 infected patients, flexible timetabling for staff, sustainable supply chains for equipment, and the development of ICU triage protocols (65).

It is noteworthy, that several physicians reported that they were not formally engaged in the institutional planning and implementation phases of new policies during the pandemic (37). Thereupon, the physicians developed their own personal policies to resolve ambiguities and uncertainties in patient care and utterly disregarded the institutional protocols (66). Thus, creating major inconsistencies in the workflow leading to heterogeneous responses and outcomes among institutions. Additionally, physicians were suspicious about changing triage protocols using ambiguous or limited resources. Finally, physicians unfortunately experienced contentious conversations with patients and their families who thought that the physicians' expertise and support services were far below the proclaimed standards of care (67).

### Recommendations

We have summarized the recommendations by appraising the key findings from the literature which can be essentially pitched at micro (personal), meso (institutional) and macro (societal) levels. At the micro level, near-peer support, meditation, and mindfulness programs can enhance physicians' psychological health. Similarly, at meso level we identified the need to pay maximum attention to institutional support by creating allocation algorithms for resources, capacity, taskforce, and budgeting decisions. Likewise, group deliberations among institutional leaders and physicians to address ethical issues and operational tasks are essential. Formal involvement of physicians in institutional planning, establishing contingency plans, structured rehabilitation programs and clear policies to mitigate moral distress for physicians and support staff. Lastly at the macro level, physicians' contributions by public should be recognized and rewarded. An effective educational policy would include a meaningful role of interprofessional education and collaboration for alleviating the work-related stress (68, 69) and structured faculty development programs to train physicians for developing their skills required for coping with sentinel events (70). Such educational interventions, supplemented by specific healthcare staff mental health support programs, carry a great potential in resurrecting the process of mental health of physicians with safe doctors and better clinical outcomes (71).

### Study limitations

This research was conducted using PubMed/Medline, Web of Science, Scopus, Science Direct, CINAHL and PsycInfo. PubMed/Medline databases. Though these databases represent a great majority of the published literature in the medical field, exclusion of other databases might have skewed our research findings. Though the researchers cross verified all stages of research including articles selection, data mining and curating, and data synthesis, the possibility of subjectiveness can't be excluded.

## Conclusion

This scoping review highlights the rise in mental distress, moral injury, and a wide range of psychopathological events among practicing physicians during the COVID-19 pandemic. There was a paradigm change in the quality of patient care. A myriad of factors including workload, stress, uncertainty, lack of morality, absence of professional advocacy and authority, and dismal institutional services contributed to the deteriorating wellbeing of physicians. There was a perceived loss of professional and legislative controls thus undermining professional advocacy and equity. This research calls for the identification and resurrection of poor workplace conditions, mental and physical rehabilitation of the affected physicians, development of plans to build individual resilience, and practice shared decision-making. Lastly, medical professionals should be allowed to serve the ailing humanity independently without any economic or political influence.

## Data availability statement

The original contributions presented in the study are included in the article/Supplementary material, further inquiries can be directed to the corresponding author.

## Author contributions

SSG, PM, IL, SYG, and FR-D contributed substantially to conceiving the idea, later validated, and approved the search strategy. SSG searched the databases and developed the search strategy. PM and IL reviewed all titles and abstracts retrieved during the initial search and grouped relevant articles for possible inclusion. To ensure consistency and research quality, SYG reviewed all initially selected titles and abstracts. All authors agreed to take responsibility for the final draft. All authors contributed to the article and approved the submitted version.
