# Supplementary material for: Evaluating the impact of COVID-19 pandemic on the physicians' psychological health: A systematic scoping review
Source: Front Med (Lausanne). 2023 Mar 28;10:1071537. doi: 10.3389/fmed.2023.1071537 (PMC10086257; doi:10.3389/fmed.2023.1071537)
Supplement: Supplementary file 1 [file Data_Sheet_1.docx]

**Appendix I:**

**PubMed – (n=128) 17^th^ June 2021**

Search: **((Physicians) AND ((((Covid 19) OR (Sars CoV2)) OR (acute respiratory distress syndrome covid-19)) OR (2019-nCoV))) AND ((((moral injury) OR (moral distress)) OR (moral conflict)) OR (ethical dilemmas))**

("physician s"[All Fields] OR "physicians"[MeSH Terms] OR "physicians"[All Fields] OR "physician"[All Fields] OR "physicians s"[All Fields]) AND ("covid 19"[All Fields] OR "covid 19"[MeSH Terms] OR "covid 19 vaccines"[All Fields] OR "covid 19 vaccines"[MeSH Terms] OR "covid 19 serotherapy"[All Fields] OR "covid 19 serotherapy"[Supplementary Concept] OR "covid 19 nucleic acid testing"[All Fields] OR "covid 19 nucleic acid testing"[MeSH Terms] OR "covid 19 serological testing"[All Fields] OR "covid 19 serological testing"[MeSH Terms] OR "covid 19 testing"[All Fields] OR "covid 19 testing"[MeSH Terms] OR "sars cov 2"[All Fields] OR "sars cov 2"[MeSH Terms] OR "severe acute respiratory syndrome coronavirus 2"[All Fields] OR "ncov"[All Fields] OR "2019 ncov"[All Fields] OR (("coronavirus"[MeSH Terms] OR "coronavirus"[All Fields] OR "cov"[All Fields]) AND 2019/11/01:3000/12/31[Date - Publication]) OR ("Sars"[All Fields] AND "CoV2"[All Fields]) OR (("respiratory distress syndrome"[MeSH Terms] OR ("respiratory"[All Fields] AND "distress"[All Fields] AND "syndrome"[All Fields]) OR "respiratory distress syndrome"[All Fields] OR ("acute"[All Fields] AND "respiratory"[All Fields] AND "distress"[All Fields] AND "syndrome"[All Fields]) OR "acute respiratory distress syndrome"[All Fields]) AND ("covid 19"[All Fields] OR "covid 19"[MeSH Terms] OR "covid 19 vaccines"[All Fields] OR "covid 19 vaccines"[MeSH Terms] OR "covid 19 serotherapy"[All Fields] OR "covid 19 serotherapy"[Supplementary Concept] OR "covid 19 nucleic acid testing"[All Fields] OR "covid 19 nucleic acid testing"[MeSH Terms] OR "covid 19 serological testing"[All Fields] OR "covid 19 serological testing"[MeSH Terms] OR "covid 19 testing"[All Fields] OR "covid 19 testing"[MeSH Terms] OR "sars cov 2"[All Fields] OR "sars cov 2"[MeSH Terms] OR "severe acute respiratory syndrome coronavirus 2"[All Fields] OR "ncov"[All Fields] OR "2019 ncov"[All Fields] OR (("coronavirus"[MeSH Terms] OR "coronavirus"[All Fields] OR "cov"[All Fields]) AND 2019/11/01:3000/12/31[Date - Publication]))) OR ("sars cov 2"[MeSH Terms] OR "sars cov 2"[All Fields] OR "2019 ncov"[All Fields])) AND ("stress disorders, post traumatic"[MeSH Terms] OR ("stress"[All Fields] AND "disorders"[All Fields] AND "post traumatic"[All Fields]) OR "post-traumatic stress disorders"[All Fields] OR ("moral"[All Fields] AND "injury"[All Fields]) OR "moral injury"[All Fields] OR (("morale"[MeSH Terms] OR "morale"[All Fields] OR "morales"[All Fields] OR "moralism"[All Fields] OR "moralities"[All Fields] OR "moralization"[All Fields] OR "moralize"[All Fields] OR "moralized"[All Fields] OR "moralizing"[All Fields] OR "morally"[All Fields] OR "morals"[MeSH Terms] OR "morals"[All Fields] OR "moral"[All Fields] OR "morality"[All Fields]) AND ("distress"[All Fields] OR "distressed"[All Fields] OR "distresses"[All Fields] OR "distressful"[All Fields] OR "distressing"[All Fields])) OR (("morale"[MeSH Terms] OR "morale"[All Fields] OR "morales"[All Fields] OR "moralism"[All Fields] OR "moralities"[All Fields] OR "moralization"[All Fields] OR "moralize"[All Fields] OR "moralized"[All Fields] OR "moralizing"[All Fields] OR "morally"[All Fields] OR "morals"[MeSH Terms] OR "morals"[All Fields] OR "moral"[All Fields] OR "morality"[All Fields]) AND ("conflict s"[All Fields] OR "conflict, psychological"[MeSH Terms] OR ("conflict"[All Fields] AND "psychological"[All Fields]) OR "psychological conflict"[All Fields] OR "conflict"[All Fields] OR "conflicting"[All Fields] OR "conflicts"[All Fields] OR "conflicted"[All Fields] OR "conflictive"[All Fields])) OR (("ethic s"[All Fields] OR "ethicality"[All Fields] OR "ethically"[All Fields] OR "ethics"[MeSH Terms] OR "ethics"[All Fields] OR "ethic"[All Fields] OR "ethics"[MeSH Subheading] OR "morals"[MeSH Terms] OR "morals"[All Fields] OR "ethical"[All Fields]) AND ("dilemma"[All Fields] OR "dilemmas"[All Fields])))

**Translations**

**Physicians:** "physician's"[All Fields] OR "physicians"[MeSH Terms] OR "physicians"[All Fields] OR "physician"[All Fields] OR "physicians's"[All Fields]

**Covid 19:** ("COVID-19" OR "COVID-19"[MeSH Terms] OR "COVID-19 Vaccines" OR "COVID-19 Vaccines"[MeSH Terms] OR "COVID-19 serotherapy" OR "COVID-19 serotherapy"[Supplementary Concept] OR "COVID-19 Nucleic Acid Testing" OR "covid-19 nucleic acid testing"[MeSH Terms] OR "COVID-19 Serological Testing" OR "covid-19 serological testing"[MeSH Terms] OR "COVID-19 Testing" OR "covid-19 testing"[MeSH Terms] OR "SARS-CoV-2" OR "sars-cov-2"[MeSH Terms] OR "Severe Acute Respiratory Syndrome Coronavirus 2" OR "NCOV" OR "2019 NCOV" OR (("coronavirus"[MeSH Terms] OR "coronavirus" OR "COV") AND 2019/11/01[PDAT] : 3000/12/31[PDAT]))

**acute respiratory distress syndrome:** "respiratory distress syndrome"[MeSH Terms] OR ("respiratory"[All Fields] AND "distress"[All Fields] AND "syndrome"[All Fields]) OR "respiratory distress syndrome"[All Fields] OR ("acute"[All Fields] AND "respiratory"[All Fields] AND "distress"[All Fields] AND "syndrome"[All Fields]) OR "acute respiratory distress syndrome"[All Fields]

**covid-19:** ("COVID-19" OR "COVID-19"[MeSH Terms] OR "COVID-19 Vaccines" OR "COVID-19 Vaccines"[MeSH Terms] OR "COVID-19 serotherapy" OR "COVID-19 serotherapy"[Supplementary Concept] OR "COVID-19 Nucleic Acid Testing" OR "covid-19 nucleic acid testing"[MeSH Terms] OR "COVID-19 Serological Testing" OR "covid-19 serological testing"[MeSH Terms] OR "COVID-19 Testing" OR "covid-19 testing"[MeSH Terms] OR "SARS-CoV-2" OR "sars-cov-2"[MeSH Terms] OR "Severe Acute Respiratory Syndrome Coronavirus 2" OR "NCOV" OR "2019 NCOV" OR (("coronavirus"[MeSH Terms] OR "coronavirus" OR "COV") AND 2019/11/01[PDAT] : 3000/12/31[PDAT]))

**2019-nCoV:** "sars-cov-2"[MeSH Terms] OR "sars-cov-2"[All Fields] OR "2019 ncov"[All Fields]

**moral injury:** "stress disorders, post-traumatic"[MeSH Terms] OR ("stress"[All Fields] AND "disorders"[All Fields] AND "post-traumatic"[All Fields]) OR "post-traumatic stress disorders"[All Fields] OR ("moral"[All Fields] AND "injury"[All Fields]) OR "moral injury"[All Fields]

**moral:** "morale"[MeSH Terms] OR "morale"[All Fields] OR "morales"[All Fields] OR "moralism"[All Fields] OR "moralities"[All Fields] OR "morality's"[All Fields] OR "moralization"[All Fields] OR "moralize"[All Fields] OR "moralized"[All Fields] OR "moralizing"[All Fields] OR "morally"[All Fields] OR "morals"[MeSH Terms] OR "morals"[All Fields] OR "moral"[All Fields] OR "morality"[All Fields]

**distress:** "distress"[All Fields] OR "distressed"[All Fields] OR "distresses"[All Fields] OR "distressful"[All Fields] OR "distressing"[All Fields]

**moral:** "morale"[MeSH Terms] OR "morale"[All Fields] OR "morales"[All Fields] OR "moralism"[All Fields] OR "moralities"[All Fields] OR "morality's"[All Fields] OR "moralization"[All Fields] OR "moralize"[All Fields] OR "moralized"[All Fields] OR "moralizing"[All Fields] OR "morally"[All Fields] OR "morals"[MeSH Terms] OR "morals"[All Fields] OR "moral"[All Fields] OR "morality"[All Fields]

**conflict:** "conflict's"[All Fields] OR "conflict, psychological"[MeSH Terms] OR ("conflict"[All Fields] AND "psychological"[All Fields]) OR "psychological conflict"[All Fields] OR "conflict"[All Fields] OR "conflicting"[All Fields] OR "conflicts"[All Fields] OR "conflicted"[All Fields] OR "conflictive"[All Fields]

**ethical:** "ethic's"[All Fields] OR "ethicality"[All Fields] OR "ethically"[All Fields] OR "ethics"[MeSH Terms] OR "ethics"[All Fields] OR "ethic"[All Fields] OR "ethics"[Subheading] OR "morals"[MeSH Terms] OR "morals"[All Fields] OR "ethical"[All Fields]

**dilemmas:** "dilemma"[All Fields] OR "dilemmas"[All Fields]

**Web of science (n=27)**

| **Set** | **Results** | **Save History / Create Alert Open Saved History** | **Edit Sets** | **Combine Sets**  **AND  OR**  **Combine** | **Delete Sets**  **Select All** **Delete** |
| --- | --- | --- | --- | --- | --- |
| 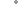 | | | | | |
| # 12 | [**27**](http://0x10irvst.y.http.apps.webofknowledge.com.proxy.rcsibahrainelibrary.com/summary.do?product=WOS&doc=1&qid=12&SID=5BAPvL1NjEAwHaGU6uO&search_mode=CombineSearches&update_back2search_link_param=yes) | #11 AND #10 AND #1  *Indexes=SCI-EXPANDED, CPCI-S Timespan=All years* | [Edit](http://0x10irvst.y.http.apps.webofknowledge.com.proxy.rcsibahrainelibrary.com/WOS_AdvancedSearch_input.do?product=WOS&SID=5BAPvL1NjEAwHaGU6uO&search_mode=AdvancedSearch&replaceSetId=12&editState=init) |  |  |
| 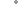 | | | | | |
| # 11 | [**7,150**](http://0x10irvst.y.http.apps.webofknowledge.com.proxy.rcsibahrainelibrary.com/summary.do?product=WOS&doc=1&qid=11&SID=5BAPvL1NjEAwHaGU6uO&search_mode=CombineSearches&update_back2search_link_param=yes) | #9 OR #8 OR #7 OR #6  *Indexes=SCI-EXPANDED, CPCI-S Timespan=All years* | [Edit](http://0x10irvst.y.http.apps.webofknowledge.com.proxy.rcsibahrainelibrary.com/WOS_AdvancedSearch_input.do?product=WOS&SID=5BAPvL1NjEAwHaGU6uO&search_mode=AdvancedSearch&replaceSetId=11&editState=init) |  |  |
| 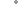 | | | | | |
| # 10 | [**93,905**](http://0x10irvst.y.http.apps.webofknowledge.com.proxy.rcsibahrainelibrary.com/summary.do?product=WOS&doc=1&qid=10&SID=5BAPvL1NjEAwHaGU6uO&search_mode=CombineSearches&update_back2search_link_param=yes) | #5 OR #4 OR #3 OR #2  *Indexes=SCI-EXPANDED, CPCI-S Timespan=All years* | [Edit](http://0x10irvst.y.http.apps.webofknowledge.com.proxy.rcsibahrainelibrary.com/WOS_AdvancedSearch_input.do?product=WOS&SID=5BAPvL1NjEAwHaGU6uO&search_mode=AdvancedSearch&replaceSetId=10&editState=init) |  |  |
| 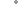 | | | | | |
| # 9 | [**4,479**](http://0x10irvst.y.http.apps.webofknowledge.com.proxy.rcsibahrainelibrary.com/summary.do?product=WOS&doc=1&qid=9&SID=5BAPvL1NjEAwHaGU6uO&search_mode=GeneralSearch&update_back2search_link_param=yes) | **TOPIC:** (ethical dilemmas)  *Indexes=SCI-EXPANDED, CPCI-S Timespan=All years* | [Edit](http://0x10irvst.y.http.apps.webofknowledge.com.proxy.rcsibahrainelibrary.com/WOS_AdvancedSearch_input.do?product=WOS&SID=5BAPvL1NjEAwHaGU6uO&search_mode=AdvancedSearch&replaceSetId=9&editState=init) |  |  |
| 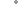 | | | | | |
| # 8 | [**1,465**](http://0x10irvst.y.http.apps.webofknowledge.com.proxy.rcsibahrainelibrary.com/summary.do?product=WOS&doc=1&qid=8&SID=5BAPvL1NjEAwHaGU6uO&search_mode=GeneralSearch&update_back2search_link_param=yes) | **TOPIC:** (moral conflict)  *Indexes=SCI-EXPANDED, CPCI-S Timespan=All years* | [Edit](http://0x10irvst.y.http.apps.webofknowledge.com.proxy.rcsibahrainelibrary.com/WOS_AdvancedSearch_input.do?product=WOS&SID=5BAPvL1NjEAwHaGU6uO&search_mode=AdvancedSearch&replaceSetId=8&editState=init) |  |  |
| 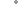 | | | | | |
| # 7 | [**1,425**](http://0x10irvst.y.http.apps.webofknowledge.com.proxy.rcsibahrainelibrary.com/summary.do?product=WOS&doc=1&qid=7&SID=5BAPvL1NjEAwHaGU6uO&search_mode=GeneralSearch&update_back2search_link_param=yes) | **TOPIC:** (moral distress)  *Indexes=SCI-EXPANDED, CPCI-S Timespan=All years* | [Edit](http://0x10irvst.y.http.apps.webofknowledge.com.proxy.rcsibahrainelibrary.com/WOS_AdvancedSearch_input.do?product=WOS&SID=5BAPvL1NjEAwHaGU6uO&search_mode=AdvancedSearch&replaceSetId=7&editState=init) |  |  |
| 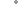 | | | | | |
| # 6 | [**411**](http://0x10irvst.y.http.apps.webofknowledge.com.proxy.rcsibahrainelibrary.com/summary.do?product=WOS&doc=1&qid=6&SID=5BAPvL1NjEAwHaGU6uO&search_mode=GeneralSearch&update_back2search_link_param=yes) | **TOPIC:** (moral injury)  *Indexes=SCI-EXPANDED, CPCI-S Timespan=All years* | [Edit](http://0x10irvst.y.http.apps.webofknowledge.com.proxy.rcsibahrainelibrary.com/WOS_AdvancedSearch_input.do?product=WOS&SID=5BAPvL1NjEAwHaGU6uO&search_mode=AdvancedSearch&replaceSetId=6&editState=init) |  |  |
| 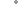 | | | | | |
| # 5 | [**1,329**](http://0x10irvst.y.http.apps.webofknowledge.com.proxy.rcsibahrainelibrary.com/summary.do?product=WOS&doc=1&qid=5&SID=5BAPvL1NjEAwHaGU6uO&search_mode=GeneralSearch&update_back2search_link_param=yes) | **TOPIC:** (2019-nCoV)  *Indexes=SCI-EXPANDED, CPCI-S Timespan=All years* | [Edit](http://0x10irvst.y.http.apps.webofknowledge.com.proxy.rcsibahrainelibrary.com/WOS_AdvancedSearch_input.do?product=WOS&SID=5BAPvL1NjEAwHaGU6uO&search_mode=AdvancedSearch&replaceSetId=5&editState=init) |  |  |
| 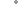 | | | | | |
| # 4 | [**2,381**](http://0x10irvst.y.http.apps.webofknowledge.com.proxy.rcsibahrainelibrary.com/summary.do?product=WOS&doc=1&qid=4&SID=5BAPvL1NjEAwHaGU6uO&search_mode=GeneralSearch&update_back2search_link_param=yes) | **TOPIC:** (acute respiratory distress syndrome covid-19)  *Indexes=SCI-EXPANDED, CPCI-S Timespan=All years* | [Edit](http://0x10irvst.y.http.apps.webofknowledge.com.proxy.rcsibahrainelibrary.com/WOS_AdvancedSearch_input.do?product=WOS&SID=5BAPvL1NjEAwHaGU6uO&search_mode=AdvancedSearch&replaceSetId=4&editState=init) |  |  |
| 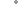 | | | | | |
| # 3 | [**1,578**](http://0x10irvst.y.http.apps.webofknowledge.com.proxy.rcsibahrainelibrary.com/summary.do?product=WOS&doc=1&qid=3&SID=5BAPvL1NjEAwHaGU6uO&search_mode=GeneralSearch&update_back2search_link_param=yes) | **TOPIC:** (Sars CoV2)  *Indexes=SCI-EXPANDED, CPCI-S Timespan=All years* | [Edit](http://0x10irvst.y.http.apps.webofknowledge.com.proxy.rcsibahrainelibrary.com/WOS_AdvancedSearch_input.do?product=WOS&SID=5BAPvL1NjEAwHaGU6uO&search_mode=AdvancedSearch&replaceSetId=3&editState=init) |  |  |
| 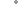 | | | | | |
| # 2 | [**93,247**](http://0x10irvst.y.http.apps.webofknowledge.com.proxy.rcsibahrainelibrary.com/summary.do?product=WOS&doc=1&qid=2&SID=5BAPvL1NjEAwHaGU6uO&search_mode=GeneralSearch&update_back2search_link_param=yes) | **TOPIC:** (Covid 19)  *Indexes=SCI-EXPANDED, CPCI-S Timespan=All years* | [Edit](http://0x10irvst.y.http.apps.webofknowledge.com.proxy.rcsibahrainelibrary.com/WOS_AdvancedSearch_input.do?product=WOS&SID=5BAPvL1NjEAwHaGU6uO&search_mode=AdvancedSearch&replaceSetId=2&editState=init) |  |  |
| 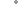 | | | | | |
| # 1 | [**297,272**](http://0x10irvst.y.http.apps.webofknowledge.com.proxy.rcsibahrainelibrary.com/summary.do?product=WOS&doc=1&qid=1&SID=5BAPvL1NjEAwHaGU6uO&search_mode=GeneralSearch&update_back2search_link_param=yes) | **TOPIC:** (Physicians)  *Indexes=SCI-EXPANDED, CPCI-S Timespan=All years* | [Edit](http://0x10irvst.y.http.apps.webofknowledge.com.proxy.rcsibahrainelibrary.com/WOS_AdvancedSearch_input.do?product=WOS&SID=5BAPvL1NjEAwHaGU6uO&search_mode=AdvancedSearch&replaceSetId=1&editState=init) |  |  |
|  |  |  |  |  |  |

**SCOPUS – (n=78)**

**Science Direct - (n= 556)**

**CINAHL – (n=90)**

**Psychinfo - (n=10)**
