# Supplementary material for: Evaluating the impact of COVID-19 pandemic on the physicians' psychological health: A systematic scoping review
Source: Front Med (Lausanne). 2023 Mar 28;10:1071537. doi: 10.3389/fmed.2023.1071537 (PMC10086257; doi:10.3389/fmed.2023.1071537)
Supplement: Supplementary file 2 [file Data_Sheet_2.docx]

**Subthemes and descriptors from theme 1 – Mental Health**

| **Subthemes in mental health** | **No. of studies** | **References** |
| --- | --- | --- |
| - **Deterrent emotions and experiences** - Cynicism or detachment from work - Cognitive stress, concentration problems, constant worrying, poor judgement, forgetfulness - Feelings of loneliness - Concentrating or decision-making difficulty due to depression - Sense of losing control - Confusion and uncertainty | 5 | (19), (20), (41), (42), (43) |
| - **Emotional distress from specific clinical situations** - Grief from witnessing patients suffer in silence - Difficulty in performing according to patient’s best interest due to imposed logistical barriers - Inability to see one’s own family - Trauma due to large number of deaths - Disease outcome uncertainties - Lack of psychological preparation | 14 | (20), (21), (22), (28), (29), (31), (34), (35), (36), (38), (39), (44), (43), (45) |
| - **Mental exhaustion** - Burnout - Sleeping disorders - Information overload - Intense physical burden and dispensable strain of additional shifts - Worsening psychological wellbeing - Uncertainty - Compassion fatigue | 19 | (19), (24), (25), (28), (30), (29), (34), (35), (37), (38), (40), (42), (43), (44), (45) |
| - **Moral injury** - Guilt feelings and frustration - Fear of an erosion in humanity - Feeling of insecurity - Sense of hopelessness - Team’s inability to respect patient’s autonomy - Dealing with triaging questions especially at times with scarce life-saving resources | 13 | (20), (21), (23), (26), (27), (30), (29), (32), (33), (34), (35), (36), (43) |

**Subthemes and descriptors for theme 2 - Individual challenges**

| **Subthemes of personal and professional challenges** | **No. of studies** | **References** |
| --- | --- | --- |
| 1. **Personal challenges**    - General Wellbeing      - Physical exhaustion, sleep deprivation, significant functional impairment and decreased quality of life      - Prior health risks and eating disorders    - Impact on family and social life      - Fear of transmitting illness to family – felt guilt, shame and feel like ‘plague spreaders’ especially to family members with health risks      - Fear of dying away from family      - Changes in routine lifestyle, work – life balance tensions      - Staying away from home      - Lack of childcare provision    - Financial burden      - Uncompensated work and salary cuts      - Furlough, loss of employment and termination of contracts | 18 | (19), (20), (21), (22), (24), (26), (32), (34), (36), (37), (38), (40), (39), (41), (42), (43), (44), (45) |
| 1. **Professional challenges**  - Work-related concerns   - Sense of powerlessness, unpreparedness and concern for work productivity   - Increased demand for care, triaging systems, remote work, mobility opportunities and technology   - Increased workload, unfair work distribution, work during antisocial hours, lack of breaks and uncompensated overtime   - Structural distress – inadequate infrastructure, changing protocols, hierarchical tensions   - Medicolegal concerns   - Violation of individual moral codes   - Strained collegial relationships - Access to resources   - Fear of contamination made worse by limited resources   - Inadequate training and strain due to the continuous use of PPE - Training and career progression   - Feelings of reduced professional accomplishments and challenging their competence   - Information fatigue   - Need for COVID-19 training due to lack of confidence   - Lack of recognition and emphasis on academic promotion   - Possible extensions to training period   - Insufficient training in resource allocation | 23 | (19), (20), (21), (22), (24), (25), (26), (27), (28), (30), (31), (32), (34), (35), (36), (37), (38), (39), (41), (42), (43), (44), (45) |

**Subthemes and descriptors for theme 3 - decision-making**

| **Subthemes of decision – making** | **No. of studies** | **References** |
| --- | --- | --- |
| 1. **Rationing care and triaging decisions**  - Life-saving treatment options, personal protection equipment resources and staffing - Decisions made based on age, patients’ cognitive status, ethnicity and race, survivability - Risk to self - Ethical considerations | 13 | (19), (20), (22), (23), (24), (26), (28), (30), (29), (31), (32), (46), (33), (35), (34), (36), (37), (41), (38), (40), (39), (41) |
| 1. **Institutional or hierarchical impact on individual decision – making outcomes**  - Family visitation restrictions - Updates in or a lack of protocols - Powerlessness and sense of futility - Lack of clear communication from institutions | 10 | (24), (29), (31), (32), (46), (33), (34), (35), (36), (37), (38) |
| 1. **Skills and strategies** to improve decision-making  - Shared decision – making with peers, the family and other departments - Team cohesion and leadership - Adapting one’s practices and behaviors, taking initiatives, being autonomous, finding a balance between constraints and values - Problem solving skills assessed by confidence, approach-avoidance, and personal control | 5 | (29), (31), (32), (46), (39) |

**Subthemes and descriptors for theme 4 - change in patient care**

| **Subthemes of change in patient care** | **No. of studies** | **References** |
| --- | --- | --- |
| 1. **Suboptimal care provision**  - Delay and restrictions - Resource limitation and unfair allocations - Vulnerable non-COVID patients and postponement of elective and scheduled care - Focus on infection risk at the expense of quality care - Preventing families from meeting loved ones in their dying moments - Strict visitor restrictions affecting the quality of care - Redeployment to other specialties | 16 | (20), (22), (23), (24), (25), (29), (46), (33), (34), (35), (37), (38), (41), (42), (43), (44) |
| 1. **Doctor - patient interactions**  - Depersonalization (lack of compassion, impersonal responses), dehumanized interactions and inability to provide emotional support - Inability to provide clear and concise information - Inability to perform physical examination and discuss difficult conversations - Fear of contamination; limited physical contact - Use of technology, PPE, and remote care - Difficulties creating a bond with patients - Limited non-verbal communication and facial expressions, barriers to verbal communication- muffled voices, no lip reading, loss of professional distance and unusual intimacy towards patients | 10 | (19), (21), (28), (31), (32), (34), (35), (37), (39), (43) |
| 1. **Physicians' duty of care**  - A sense of sacrifice, conscientiousness, and moral responsibility - Employing ethics service to advocate for patients and considerations of patients’ autonomy, beneficence and honouring patients’ wishes - Thinking of creative ways to deliver outstanding care - Willingness to try nonevidence-based treatment plans - Provision of in-person care even during PPE scarcity | 5 | (26), (30), (31), (35), (36) |

**Subthemes and descriptors for theme 5 - support services**

| **Subthemes in support services** | **No. of studies** | **References** |
| --- | --- | --- |
| **1.** **Near-peer support**   - Various individual-centred interventions to tackle the emotional toll:   - Self-coping strategies (e.g., positive reframing, active coping, planning, acceptance)   - Peer support group sessions (guidance consensual validation instillation of hope, peer-learning, psychoeducational resources   - Counselling sessions   - Virtual social media-based support platforms | 9 | (20), (22), (23), (28), (34), (36), (37), (41), (44) |
| **2.** **Institutional interventions**   - - To aid in wellbeing:     - Management and administrative     - Feedback reporting mechanisms   - To aid in decision – making:   - Ethics consult service   - Shared policy making   - Multi-professional crisis management | 8 | (20), (22), (26), (30), (31), (32), (35), (38) |
